# Socio-economic assessment of dog population management systems: a scoping review

**DOI:** 10.3389/fvets.2025.1519913

**Published:** 2025-01-20

**Authors:** Rabina Ghimire, Parimala Mohanty, Elly Hiby, Andrew Larkins, Salome Dürr, Sonja Hartnack

**Affiliations:** ^1^Section of Epidemiology, Vetsuisse Faculty, University of Zurich, Zurich, Switzerland; ^2^Jyoti and Bhupat Mehta School of Health Sciences and Technology, Indian Institute of Technology, Guwahati, Assam, India; ^3^International Companion Animal Management (ICAM) Coalition, Cambridge, United Kingdom; ^4^School of Medical, Molecular and Forensic Sciences, Murdoch University, Perth, WA, Australia; ^5^Centre for Biosecurity and One Health, Harry Butler Institute, Murdoch University, Perth, WA, Australia; ^6^Veterinary Public Health Institute, Vetsuisse Faculty, University of Bern, Bern, Switzerland

**Keywords:** dog, population management, dog population management (DPM) services, cost, benefit, socio-economic impact

## Abstract

**Introduction:**

Dog Population Management (DPM) systems primarily aim to reduce the free-roaming dog population, improve the health and welfare of humans and dogs, and foster their peaceful coexistence. A key challenge to resource allocation and evidence-based policy making in DPM is the rare evaluation of the associated socio-economic impacts. This scoping review identifies, maps, and summarizes published parameters and methods on the socio-economic aspect of DPM systems.

**Methods:**

Following PRISMA-ScR guidelines, and with a protocol registered on the Open Science Framework, this review explores (i) types of DPM services, (ii) types of parameters (intervention, impact, monetized, or non-monetized), (iii) methodological approaches (such as cost–benefit or cost-effectiveness analysis), and (iv) gaps and challenges in socio-economic DPM assessments. Relevant publications were identified through a systematic search of PubMed, Embase, Scopus, and Web of Science.

**Results:**

Our review identified 14 out of more than 7,200 studies indicating the limitation of socio-economic data associated with DPM systems. The studies revealed diverse approaches to DPM, sterilization being the most frequently used service, often combined with vaccination and community awareness. Culling was also used by several studies as a DPM intervention, though considered unethical. The review highlighted a range of intervention, impact, and monetary parameters to evaluate the economics of DPM systems, demonstrating the complexity and varied scope of the services. Varied categorizations of the dog population were observed, making comparative evaluation challenging. Economic methods such as cost–benefit and cost-effectiveness analyses were observed, identifying several associated economic metrics. Studies highlighted gaps mostly related to data availability and accessibility.

**Conclusion:**

The limitations of socio-economic data arise from a lack of standardized methodologies across regions and contexts and limited data collection efforts. Prioritizing systematic collection of data on costs, benefits and social impacts allows for a more robust analysis of DPM systems. Developing tools and standardized reporting methods would further facilitate consistent evaluation of impacts, efficient resource allocation and evidence-based policy making to implement the most cost-effective DPM systems.

**Systematic review registration:**

DOI: 10.17605/OSF.IO/NHE3X

## 1 Introduction

Dogs have been an integral component of human communities and played diverse roles, often serving as companions or working animals, including offering household or livestock protection (1, 2). However, cultural attitudes toward dogs vary; while they are treated as beloved family members in some communities, in others, they may face neglect, hunting, or culling (3, 4). There is a concern as dogs can act as a reservoir and vector of various zoonotic diseases such as rabies, echinococcosis, and leishmaniasis, among others (5–8). Dog-mediated rabies as a public health burden is estimated to cause about 59,000 human deaths per year worldwide, with transmission primarily through bites of free-roaming dogs, accounting for over 95% of infections (5). Beyond the risk of transmission of zoonotic diseases, free-roaming dogs also pose public health or safety issues by causing dog bites, road accidents, chasing people, or producing fecal and noise pollution. In addition, free-roaming dogs can affect wildlife, livestock, and other animals and birds by predation, competition for resources or disease transmission, such as canine distemper or neosporosis (6, 9–11).

Dog population management (DPM) is intended to reduce the number of unwanted dogs, improve the health and welfare of dog populations, associated problems, and foster peaceful coexistence with dogs, humans and the environment (12–14). Humane DPM focuses on maximizing advantages for both dogs and human communities while avoiding the practice of dog killing (14). A DPM system is a comprehensive programme of DPM services (also called ‘measures’). These include dog sterilization (spaying or neutering) campaigns, identification and registration of dogs, vaccinations and parasite control, dog rehoming and adoption, promotion of responsible ownership, and control of commercial breeding and sale among other initiatives (15). The primary objectives of a DPM system are to reduce the free-roaming dog population, minimize risks to public health and safety, and reduce nuisance caused by free-roaming dogs. Additionally, DPM systems seek to improve the health and welfare of humans and dogs by fostering human-animal interactions, enhancing mental well-being for dog owners, and promoting better care for dogs through vaccination, sterilization, or reduced abandonment (12). DPM systems, thus, manage the dog population and consequently support other initiatives such as rabies control by increasing and sustaining vaccination coverage and improving access of the population to surveillance as well as the management of other zoonoses and diseases (12, 16). Dog population management has evolved over time. While culling, particularly through poisoning, shooting, or beating has been deemed unethical, it continues to be implemented in some regions as part of policies to manage free-roaming dog populations (17). However, it does not include the humane termination of animal’s life to alleviate suffering and protect its welfare (18). In contrast, more recent strategies emphasize humane approaches, such as sterilization and vaccination to manage dog populations humanely and effectively (16, 19).

DPM has a social dynamic, influenced by various human behaviors, socio-economic conditions, and cultural norms, which differ across areas. These factors contribute to differences in the implementation of DPM systems, as unique dog population dynamics and ownership practices shape how dogs, often referred to as ‘community dogs’ are managed within local communities (14, 20). Assessing socio-economic conditions is crucial for tailoring DPM service provision to increase accessibility (14, 15), particularly in areas where social determinants such as education, culture, income and living conditions may limit the resources for effective dog management (9, 21). For example, countries like India and Mexico face challenges due to their large free-roaming dog populations (22, 23) and limited resources, which makes basic control measures difficult to implement. In contrast, European countries like Italy have more structured and regulated DPM systems that include comprehensive policies, mandatory dog registration, sterilization and vaccination (24). Technological advancements also shape modern DPM practices, introducing innovations like microchipping and new sterilization techniques (9, 25, 26). Furthermore, the human-animal bond influences DPM strategies, as strong relationships between humans and dogs encourage community engagement, leading to more sustainable and effective DPM systems, emphasizing the need for community involvement and stakeholder engagement in DPM initiatives.

Economic analysis supports the reality of making decisions related to resource allocation in the context of scarce resources. They evaluate the immediate and long-term costs and benefits of interventions, as well as their outcomes over time, using various scenarios and economic metrics to assess the efficiency and impact of each DPM service (27). A range of economic assessment methods, based on economic theories, are available, with Cost Benefit Analysis (CBA) and Cost-Effectiveness Analysis (CEA) being the most common to obtain various economic metrics (Table 1) (28). These economic analyses provide a framework for decision-making by offering a comparative overview of the different methods and hence to identify the most economical programs to assist in the allocation of resources and development of government DPM policy. Additionally, non-monetary measures, such as the number of animals sterilized or more complex health measures including disability-adjusted life years (DALYs) and adjusted DALYs (zDALYs) for zoonotic diseases, are also used to evaluate the impact of diseases or health interventions on human and animal populations (29).

**Table 1:** Economic terminologies and definitions.

	Definition and metrics
Cost–benefit Analysis (CBA)	Comparison of costs and benefits of an intervention to determine the expected net benefits, providing the metrics in monetary units (59). Expressed as BCR, NPV or IRR
Benefit cost ratio (BCR)	Compares total expected benefits with total expected costs. BCR = Total Benefits/Total Costs (if BCR >1, benefits of the intervention outweigh the costs)
Net Present Value (NPV)	Compares a present value of cash inflow over a present value of cash outflow (60), and equals to the sum of discounted cash inflows less the sum of discounted cash outflows
Internal Rate of Return (IRR)	Estimates the profitability of interventions, defined as the discount rate that makes the NPV from a particular intervention equal to zero
Cost-effectiveness analysis (CEA)	Measures outcome or effectiveness in naturally occurring health or intervention related units (61)
Cost effectiveness ratio (CER)	Measure of CEA which expresses the price per effectiveness unit (e.g., price per DALYs averted) (28) CER = Cost of intervention/ Effectiveness/outcome of intervention
Discounting	Interest rate that adjusts the future costs and benefits to make them comparable to present values accounting for time value of money (59)

The socio-economics of DPM systems, though important, is rarely evaluated, and there is limited published evidence available. The absence of this information on the socio-economic aspects of different DPM services and their efficacy restricts assessments. Therefore, it is crucial to have an overview of what past efforts do exist, to understand the economics of the DPM system as far as they are currently understood. This scoping review is conducted to synthesize evidence that assesses the socio-economic considerations in the DPM systems.

The aim of this scoping review is to identify, map, and summarize the published methods and parameters on the socio-economic aspects of DPM systems. The primary outcome is to determine available evidence, identify the current gaps, and establish the need for enabling future socio-economic assessment. Furthermore, the outputs based on the scoping review will contribute to determine the types of data needed and methodologies best suited for future socio-economic assessments to develop recommendations for important policy decisions and community-based initiatives for effective DPM.

## 2 Methods

### 2.1 Protocol and registration

This review was directed to summarizing the published evidence demonstrating the socio-economic considerations of the DPM systems. It was based on the guidelines of Preferred Reporting Items for Systematic Reviews and Meta-Analysis (PRISMA) statement extension for scoping review (PRISMA-ScR) (30). It was conducted according to the methodological framework developed by Arksey and O’Malley (31), following the Joanna Briggs Institute (JBI) manual for evidence synthesis. This includes (i) identifying the research question; (ii) identifying relevant studies; (iii) study selection; (iv) charting the data; and (v) collating, summarizing and reporting the results (32). The final protocol was registered with the Open Science Framework (OSF) (33).

### 2.2 Review questions

The review questions focused on the socio-economic assessment of the DPM systems.

a. Which DPM services were considered, and which dog populations were targeted?
b. In which geographical locations were these DPM services implemented?
c. What parameters (intervention, impact, monetary or non-monetary) are available for the socio-economic assessment of DPM services?
d. What is the research approach (retrospective, prospective or modeling)?
e. What methods and metrics are used for assessing the socio-economic impact of DPM systems/services?

- Cost–benefit analysis (CBA), cost-effectiveness analysis (CEA) or others.
- Was discounting rate applied in the economic assessment (if yes, what rate was chosen)?
- What are the economic metrics for the assessment (Benefit Cost Ratio (BCR), Net Present Value (NPV), Cost-effectiveness Ratio (CER) or others)?

f. What are the other impacts of the economic costs and benefits on the society?

- Values attributed to dogs and their health.
- DALYs averted.
- Reduction in disease transmission.
- Improvement in community/safety perceptions.

g. Do these DPM services have an intersection with other human or animal health activities? If yes, which interventions?
h. What gaps and challenges were identified in the study regarding the socio-economic assessment of DPM systems?

### 2.3 Study screening and selection

#### 2.3.1 Information sources and search strategy

With the help of a professional librarian, the search strategies were identified. For a literature search, the following databases were searched for peer-reviewed publications and reports: PubMed, Embase, Scopus, and Web of Science All Databases (Web of Science Core Collection, Biosis Citation Index, Biosis Previews, Medline, Zoological Record). No date restrictions were applied. Additional gray literature was also identified from organizations working on DPM or pet welfare utilizing contacts from the authors as well as the International Companion Animal Management Coalition (ICAM) and authors. Also, the websites of individual international non-governmental organizations working on DPM were searched for data not published in the peer-reviewed papers. Potentially relevant references from selected studies were also searched to ensure that the key papers were not missed.

The search strategy (Annex 1) developed was the combination of synonyms of “dog” and “population management” while using a proximity search between seven words.

#### 2.3.2 Study selection

Inclusion criteria were as follows: (i) consider the DPM services (sterilization or reproduction control, dog identification or registration, sheltering and adoption) or culling or dog removal as part of the interventions, (ii) demonstrate the economics (costs, benefits, or effectiveness in monetary or non-monetary terms) of DPM services (include either sterilization, or identification or adoption and sheltering) or culling, and (iii) be a primary literature (original research papers or case studies).

Exclusion criteria were defined as follows: (i) no evidence of DPM, (ii) does not consider the socio-economic factors associated with DPM systems, (iii) is not a primary research source (the research was a review or commentary), and (iv) only shows the economic assessment of dog vaccination or parasite control without a link to other DPM services.

#### 2.3.3 Selection of data

All the data retrieved were combined in Endnote and deduplication was performed. These data were then imported into Rayyan (34) and the remaining duplicates were detected and removed. The data were screened in two phases. First, the title and abstracts were screened by reviewers independently (RG, PM, and SH) in a blinded manner according to the relevance to our research questions, assigning the values “include,” “maybe” or “exclude.” In cases where there were conflicts, such as differences in decisions between “include,” “exclude,” or “maybe,” among reviewers, consensus was reached through discussion.

The data selected from the abstract and title screening that met the inclusion criteria were then further screened for a full-text review, again by a minimum of two reviewers (RG, PM, and SH). In cases of conflicts, these were resolved through discussion among reviewers. Papers selected from the full-text review, significant papers identified from the retrospective search of the included literature, and publications/reports from the gray literature were then included as the final data for review.

### 2.4 Charting the data

From the papers selected for a full-text review and other relevant reports, data were extracted using a data extraction sheet in MS Excel.

The data items extracted in the data extraction sheet are as follows:

- Bibliometric details: Title, authors, year of publication.
- Study details: geographical location, timeframe, objectives, research approach and focus of the study.
- DPM services considered: Sterilization/fertility control, dog identification and registration, dog adoption/sheltering, dog vaccination, community awareness and education, and culling.
- Intersection with other human or animal health interventions.
- Quantitative parameters: Intervention parameters and impact parameters.
- Monetary parameters: costs of the services, operational, staff and other costs, benefits from the DPM systems.
- Non-monetary parameters.
- Economic methodologies and metrics.
- Gaps and challenges in the economic assessment of DPM presented in the study.

Data charting and synthesis of results helped to identify key parameters and methodologies of DPM economics.

### 2.5 Critical appraisal of the evidence source

Due to the scoping nature of the review, critical appraisal of the methodologies, bias assessment of the records and in-depth analysis were not performed.

### 2.6 Analysis

Utilizing review questions and data charting as guiding frameworks, a qualitative thematic analysis was conducted to synthesize key findings and themes from the literature, providing a comprehensive summary of the current state of socio-economic parameters and methods in DPM systems.

Statistical analysis and data visualization was performed using R *(Version 2024.04.2 Build 764)*. The “UpsetR” package (35) was employed to create the UpSet plot that visualized different frequencies and intersections of various DPM services across multiple studies included. R codes are attached (Annex 2).

## 3 Results

### 3.1 Screening and selection process and geographical origin of studies

**Figure 1:**
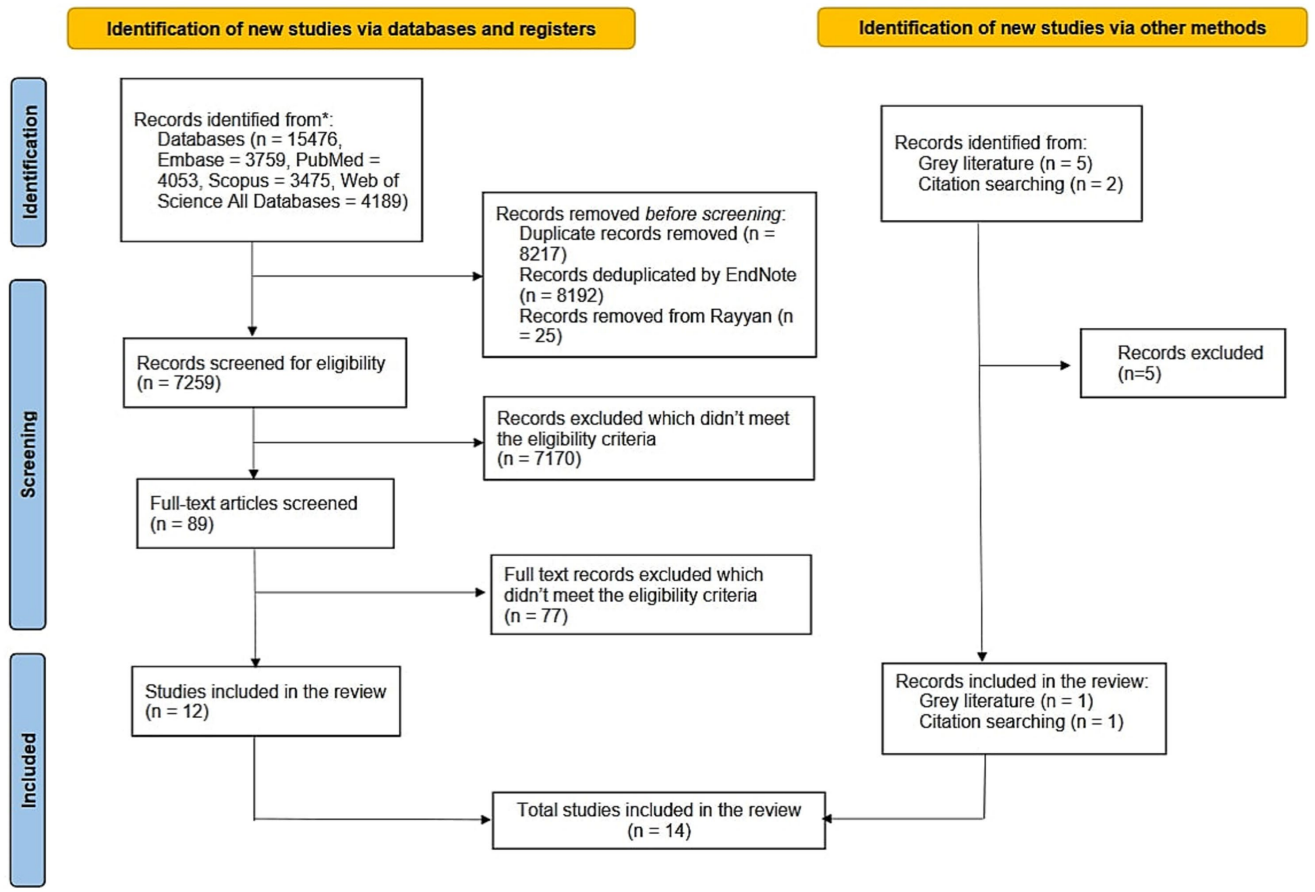
PRISMA scoping review flow diagram presenting the identified, screened, and included studies, from database searches, gray literature, and citation searches.

We identified 15,476 references from four databases (Figure 1). After deduplication in Endnote, 7,284 were imported in Rayyan. Within Rayyan, 25 duplicates were identified and removed while retaining the original references. We screened through 7,259 studies for the titles and abstracts, resulting in the selection of 89 studies that met our eligibility criteria. For the final data charting process, only 12 studies from the database search met our criteria. Additionally, one study from the gray literature search and one from the citation search also met our criteria, bringing the total number of studies included in the final review corpus to 14.

The 14 studies included in the review were published between 2006 and 2022 (Table 2). The study encompasses diverse geographical locations across multiple continents and countries including Italy (36), Ukraine (25) and North Macedonia (13) in Europe, India (37, 38), Indonesia (39, 40), the Philippines (41), Sri Lanka (42), Bhutan (43) in Asia, in Chile (44) and Brazil (45) in South America, and the United States (46). The global scope of rabies elimination efforts is discussed by Wallace et al. (47), encompassing various regions worldwide.

**Table 2:** Descriptives of the studies and Dog Population Management services with their respective methodologies.

Study	Publication date	Geographical location	Dog population	Study focus	Sterilization		Dog identification/registration	Sheltering capacity	Adoption and rehoming
					Sterilize	Sterilize and vaccinate			
Smith et al., 2022 (25)	2022	Ukraine, Europe	Owned and unowned free-roaming (restricted), owned home-restricted, and unowned shelter dogs	DPM	Yes			Yes	Yes
Ćetković et al., 2022 (13)	2022	North Macedonia, Southeast Europe	Stray dogs	DPM	Yes			Yes	
Garde et al., 2022 (44)	2022	Chile, South America	Owned and unowned free-roaming and owned confined dogs	DPM	Yes		Yes (microchipping and collars/tattoos)		Yes
Diamante et al., 2021 (41)	2021	Philippines, Southeast Asia	Free-roaming and stray dogs	Rabies		Yes			
Larkins et al., 2020 (37)	2020	India, South Asia	Roaming dogs	Rabies		Yes			
Wallace et al., 2017 (47)	2017	Global	Free-roaming (owned and unowned) and owned confined dogs	Rabies	Yes				
Dias et al., 2015 (45)	2015	Brazil, South America	Owned (confined and free-roaming) dogs	DPM	Yes				Yes
Häsler et al., 2014 (42)	2014	Sri Lanka, South Asia	Owned dogs, Roaming dogs (Unowned or community-owned, or owned)	Rabies		Yes			
Abbas et al., 2014 (38)	2014	India, South Asia	Stray dogs	Rabies	Yes^a^	Yes			
Wera et al., 2013 (39)	2013	Indonesia, Asia	Free-roaming (owned and unowned) dogs	Rabies					
Høgåsen et al., 2013 (36)	2013	Italy, Europe	Stray, kennel, block and owned (confined and free-roaming) dogs	DPM	Yes		Yes (microchipping)	Yes	Yes
Tenzin et al., 2012 (43)	2012	Bhutan, South Asia	Stray and pet dogs	Rabies		Yes (for stray dogs)			
Häsler et al., 2012 (40)	2012	Indonesia, Asia	Free-roaming (owned and unowned) and owned confined dogs	Rabies					
Poss and Everett, 2006 (46)	2006	USA, North America	Owned dogs	DPM	Yes				

a Surgical sterilization and injectable contraception.

### 3.2 Categories of dog populations

Various dog populations were identified based on ownership and movement restrictions. The categories included owned dogs, which were either confined (25, 36, 40, 44, 45, 47) or free-roaming (25, 36, 39, 42, 44, 45, 47), or pet dogs (43), unowned free-roaming (25, 39, 40, 42, 44, 47), stray (13, 38, 43), community-owned (42), shelter (25), block (36), or kennel dogs (36). Some studies only mentioned owned (42, 46) or roaming dogs (37) without further differentiation. These categorizations were taken as stated in the papers, reflecting the diverse terminologies used to describe these populations.

### 3.3 Intersection with animal/human health

Among the 14 studies from our review corpus, 6 (43%) of them had a focus on DPM services (sterilization, identification, sheltering and adoption) (13, 25, 36, 44–46) and included economic aspects, while the remaining 8 (57%) studies had a focus on rabies control (37–43, 47) and included a portion of DPM in the study (Table 1). One study also analyzed the effects of DPM on parasitic disease control, one on Leishmania (36).

### 3.4 DPM services considered

**Figure 2:**
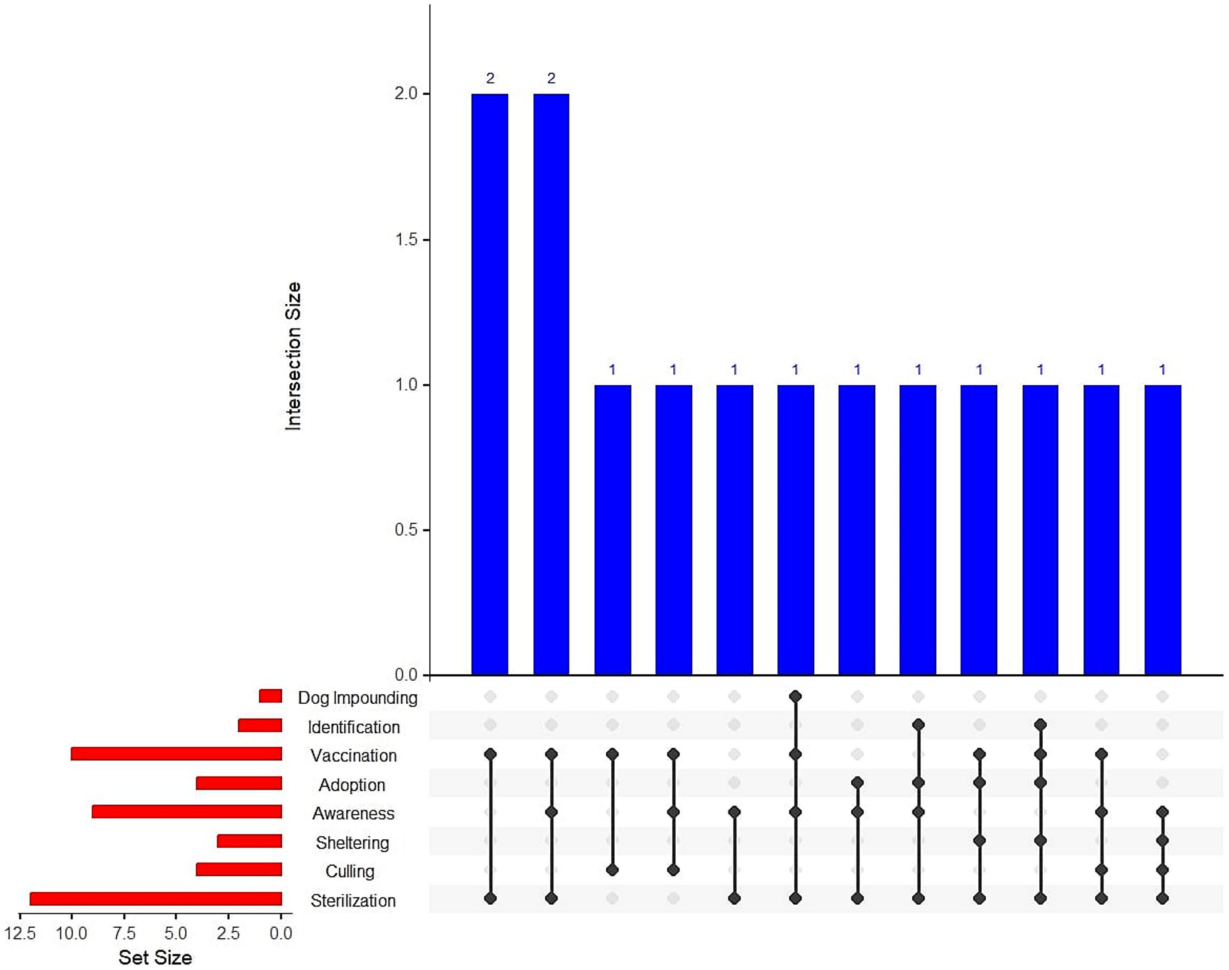
UpSet plot of DPM services and their intersection across multiple studies. The horizontal bars demonstrate DPM services and the vertical bars depict the number of studies that share specific combinations of services. The two tallest bars indicate that sterilization combined with either vaccination or awareness occurs in two studies each. The other bars represent unique combinations of services found in single studies.

We identified a range of DPM services being assessed, including sterilization, dog identification, sheltering, and adoption, as well as complementary DPM services such as vaccination, and community education programs. Culling and impounding were also used as a DPM intervention in some studies. A range of combinations of the DPM services were identified (Figure 2). Almost all the studies used sterilization, which was often combined with vaccination and/or awareness.

Among the reviewed articles, we found twelve (86%) studies that reported sterilization or fertility control as a DPM service. The studies discussed either sterilization, e.g., mobile spay-neuter clinic (46), or a combination of animal sterilization and anti-rabies vaccination termed synonymously as Catch-Sterilize-Vaccinate-Return (37) or Animal Birth Control with Anti-Rabies vaccination (38). Additionally, as a method of non-surgical sterilization, injectable contraception was also modeled for application (38).

Dog identification and registration was identified as a DPM service by only two (14%) of our retrieved studies. In Chile, identification included microchipping, tattoos, or collars with identification tags containing a unique code (44). Likewise, in Abruzzo, Italy (36), dog identification was mandated by law, hence all the dogs were microchipped and registered.

Three (21%) studies in the review (13, 25, 36) highlight dog sheltering as one of the important DPM services with a focus on increasing shelter capacity (13, 36), or use of shelters to take in relinquished dogs making them kennel dogs (36). Adoption and rehoming was identified in four studies (29%), including practices such as adoption from kennels, or rescuing and rehoming free-roaming dogs to reduce abandonment (25, 36, 44, 45).

Among our retrieved studies, ten (71%) included the canine rabies vaccinations (13, 36–43, 45, 47). Interventions included combining vaccination and sterilization (37, 38, 43) or using a combination of injectable and oral rabies vaccination for dogs (38). An annual or biannual mass vaccination program was implemented in Bhutan for both owned and stray dogs (43). Similar mass vaccination initiatives were employed for dog vaccinations in Bali and Davao (40, 41).

Community awareness and education were included as DPM services in eight (57%) studies. The community awareness interventions were focused on increasing responsible dog ownership (25, 42, 44, 47). Information and education campaigns (IEC) were conducted in schools and targeted both children and adults (25, 41, 42, 47). These campaigns also focused on raising awareness of rabies through bite prevention and promoting vaccination for dogs (38, 47). Use of local leaders, local media, and the distribution of flyers was also undertaken to promote community awareness (39, 46).

### 3.5 Dog culling

Culling was identified in four (28%) of the studies in our review (25, 39–42). The study from Flores Island, Indonesia describes culling as an intervention where dogs were killed by being beaten with sticks by the local community or culled by shooting, typically by a team that was formed by a regency administrator (39). Another study conducted in Colombo, Sri Lanka reported on culling during the baseline scenario when CO_2_ and CO poisoning were used for dog culling (42). Also, dogs killed in Bali, Indonesia using strychnine poisoning formed the baseline scenario for the study (40). Smith et al. (25) also mentioned culling as one of the DPM measures.

Likewise, in Davao city, Philippines, dog impounding was used as a measure to remove the suspected rabid dogs from the population (41). Here, impounding refers to the removal of free-roaming dogs. They are taken to the city pound and if unclaimed, euthanized within 3 days.

### 3.6 Research approach and timeframe

The studies in the review used a range of approaches, with a majority (12 studies or 86%) employing modeling techniques combined with observational or retrospective data including past data, census, and expert inputs to allow for retrospective or future projections (13, 25, 36–43, 45, 47) (Supplementary Table 1). For example, Smith et al. (25) used systemic dynamic modeling to simulate the DPM impact. Additionally, Wallace et al. (47) adopted predictive modeling approaches, combining the existing data, expert opinions and literature to forecast global resources needed for rabies elimination. Garde et al. (44) focused on observational and descriptive analysis including qualitative aspects, while Häsler et al. (42) used a mixed-methods approach integrating modeling and economic, ethical, and social assessments.

The time frame of the study also varied in the studies ranging from a brief observational span of just a 5-month period (46) to immediate durations of 5 years (42, 44), and 9 years (41), up to more extended spans covering 23 23-year period (37). Additionally, seven studies (50%) also incorporate future projections, with six (43%) extending over 10- to 20-year horizons (13, 36, 38, 40, 43, 45), or one even projecting up to a 70-year time frame (25).

### 3.7 Parameters for socio-economic assessment

Quantitative measures frequently included the intervention and impact parameters and provided concrete metrics to evaluate the outcomes of the DPM system by the studies (Supplementary Table 1).

Intervention parameters included the services of a DPM system. These parameters were either obtained through data collected during the implementation of DPM services or predicted through modeling. They included sterilization rates or number of animals sterilized (25, 37, 38, 41–47), number of adoptions or sheltering (13, 25, 36, 45), dogs identified or registered through microchipping (36, 44), tattoos or collars (36). Additional parameters encompassed the vaccination coverage or number of dogs vaccinated (36–43, 47) and the number of community awareness campaigns conducted (41, 44). In the studies where culling was a component of a DPM system, culling rates or the number of animals culled (25, 39, 40, 42) or dogs impounded (41) were also assessed.

Three papers (21%) studies in the review make references to dog population estimates. In regions of Chile where a specific estimate of human to dog ratio was not available, Garde et al. (44) offer a statistical tool to estimate this number. The use of a mark-resight survey and annual or biannual direct observation counts on foot or by motorbikes in Jaipur, India were used for the estimation of dog population (37). Similarly, in Brazil, census was used for owned dog population estimates (45).

The DPM services described in the selected publications were assessed for their societal and economic impact. The societal impacts included a reduction in the number of free-roaming dogs (13, 25, 36, 37, 42, 47), a reduction in dog bites (13, 37), and a decrease in rabies cases (37, 40, 42) and other diseases (Echinococcosis and Leishmaniasis) (13). These impacts also contributed to DALYs averted with rabies and dog bites (37, 40, 42).

Moreover, the implementation of DPM systems resulted in notable social impacts such as increased dog welfare (25, 36, 40, 42), improved community health/safety perceptions, and acceptance of the dog population (40, 42, 47), decreased nuisance index (36) and reduced traffic accidents (13). Loss of dog’s value due to culling was also mentioned (39).

Monetized parameters were especially crucial in cost–benefit analyses, with many studies detailing the direct costs associated with different DPM interventions. The review demonstrated a range of monetary parameters to assess the financial implications of the DPM system with each study highlighting variations in economic parameters based on the scope and scale of the services implemented (Supplementary Table 1).

Our review identifies a variety of monetary parameters. They were the total costs of the DPM services (13, 36, 37, 39, 41, 42, 44–47), including total cost per session (46) and annual costs (36). For studies focusing on rabies, total costs specifically referred to the cost of rabies control (37–43, 47). Detailed monetary parameters included sterilization costs (38, 41–44, 47), infrastructure and capital investment costs (infrastructure and capital) (13), sheltering and kennelling costs (36), community education and awareness costs (38, 41, 42, 44), vaccination costs (38–43, 47), staff costs (13, 25, 37, 40, 42, 44–47), and culling costs (39–41). Where studies considered impacts beyond the dog population, they often included costs related to human health such as post-exposure prophylaxis (PEP), treatment of dog bites, treatment of human rabies cases (37–40, 42, 43), and operational and administrative costs (13, 40, 42, 47). One study also considered the economic losses due to reduced tourist number (40).

From the economic perspective, most studies focused on a comparative reduction in costs rather than true monetary benefits. Cost savings were attributed to decreased dog bites, reduced PEP use (13, 37, 40), averted DALYs (37, 40, 42), reduced traffic road accidents involving dogs (13), increased adoption (36), and applying vaccination measures instead of dog culling (40). Only one study reported prospective direct revenue generation from DPM services, specifically through sheltering or housing owned dogs (13).

### 3.8 Economic methodologies and metrics

The review of the economic assessment methodologies used revealed a multitude of approaches and metrics. With CEA being the most used method for economic assessment of the DPM systems by all studies in our review (13, 25, 36–47). This was often combined with sensitivity analysis (13, 25, 36, 38–40, 42, 43, 45, 47), exploring the influence of different intervention parameters on the outcomes of the DPM systems. Cost-effectiveness ratios were frequently calculated. These included cost per dog sterilized (13, 36–38, 41, 43–47), cost per dog vaccinated (13, 36–39, 41, 43, 47), cost per dog sterilized and vaccinated (37, 38), cost per dog microchipped (36), cost per dog impounded (41), cost per dog culled (39) and cost per IEC session (41). The CER for quantifying the benefits of DPM services was commonly expressed as cost per DALYs averted (37, 42) and cost saved per dog bite prevented (38, 40, 42). CBA was used by five (36%) evidences (13, 36, 37, 40, 43) revealing economic metrics as BCR (13, 37), NPV (13, 37, 40) and Internal Rate of Return (IRR) (13). Discounting rates were accounted for in five papers (13, 37, 39, 42, 47) and varied, with some studies applying rates as low as 3% to some 6%. Some studies present both non-discounted and discounted values (37, 40).

### 3.9 Gaps and challenges identified for socio-economic assessment

As noted in many studies, gaps in data availability are a major challenge (13, 37, 41–44), particularly concerning dog populations, dog bites and disease frequency (38, 42, 43). Studies were also limited by methodological gaps (13, 37), and the need for assumptions due to insufficient baseline data, especially when modeling was employed for economic analysis for future projections of metrics and parameters (36, 41, 43, 44). Some studies encountered challenges in calculating the full costs of interventions as they did not include capital, training and equipment costs, and other indirect costs, leading to incomplete cost estimations (25, 36, 43). On a global scale, a significant challenge is the cost variability for interventions, e.g., dog vaccination across different regions and countries (47).

## 4 Discussion

This scoping review provides a comprehensive overview of studies that assess the socio-economic aspects of DPM systems. We reviewed the types of DPM services used, the methodologies employed, the populations studied, and the geographical contexts. Additionally, the review summarizes the parameters of DPM and economic methods applied along with associated metrics and identifies the gaps and challenges in the existing literature.

Despite the large number of titles and abstracts of studies (7,259) and gray literature screened, only 14 met our inclusion criteria, indicating the limitation of economic data available in DPM systems. Among them, most focused on rabies control with DPM interventions as a part of the program. This underscores the public health implications of DPM while highlighting a gap in independently addressing DPM and its economic assessment from rabies.

We identified only limited studies that assess sheltering (21%) or adoption (29%), despite their widespread use in DPM. This may partly be due to studies focusing on only one of the services delivered by an organization, e.g., sterilization and vaccination (37). However, it is important to note that the economic analysis of DPM is challenging due to a lack of a comprehensive framework that defines the role of DPM in society. A system modeling approach could significantly enhance the understanding by providing a more holistic view of DPM’s contributions to One Health. This approach could help guide organizations in identifying and collecting relevant data, enabling a clearer and more comprehensive evaluation of the societal, public health and animal welfare benefits while addressing the existing gaps in DPM impact measurement.

Additionally, the studies that included the economic aspects of DPM were published between 2006 and 2022, indicating that assessing the economic viability of DPM is a relatively new approach that is gaining recognition as relevant to understanding the importance and complexities of DPM. This reflects the increasing understanding that DPM requires a multifaceted approach including an appreciation of animal welfare, public health, and economic feasibility.

In our review, there was an inclusion of varied terminologies to describe dog populations. It is evident that these categorizations and descriptions are not consistently standardized across studies which may often lead to ambiguity. Terms such as ‘stray,’ ‘free roaming,’ ‘community owned,’ ‘owned,’ and ‘restricted’ are frequently used. While there are some definitions provided by the World Organisation for Animal Health (WOAH) (12), there are other terms such as ‘block dogs’ (36) that can still complicate the use of terminology. “Block dogs” are free-roaming dogs that reside in specific urban “blocks” within a city, similar to village dogs but adapted to urban settings. These dogs are collected, microchipped, sterilized, and vaccinated by Local Veterinary Services before being returned to their original localities, where they are cared for and managed by the community and local municipalities (36). Additionally, insights from research on rabies in India highlight similar challenges where terms like “stray,” “street” and “free-living” dog are used to reflect different societal perspectives on the life of dogs in communities (48). Here, they argue that the term “stray” reflects a Western view of dogs as “out of place” when not owned, contrasting with Indian perspectives that often recognize street dogs as legitimate cohabitants of public spaces. This lack of uniformity and inconsistency illustrates the difficulty in defining and categorizing dog populations, given their varied and complex interactions with humans and the socio-geographical context. This inconsistency complicates the comparison and analysis of DPM systems across different regional or cultural contexts.

Although culling has been historically used as a DPM intervention, the approach now has shifted to various alternative or more humane methods. Dog killing or culling has been prohibited by law in countries like Italy, Brazil or Bhutan, whilst allowing for euthanasia to avoid animal suffering (36, 43, 45). This change in the DPM approach was reported in several studies; in Colombo city, Sri Lanka, where culling with gas inhalation was replaced by sterilization and community education (42), in Bali, Indonesia, where culling with strychnine was replaced by mass rabies vaccination (40) and in Texas, USA mobile sterilization clinics were introduced to reduce relinquishment to shelters and subsequent euthanasia (46). Despite these positive advances, dog culling or removal is still practiced in some regions across Asia and Africa such as the Philippines (41), India (49), or Uganda (50) emphasizing the need for greater operational engagement and robust efforts to stop culling practices. Culling is short-lived, any population that experiences rapid reduction is swiftly restored due to increased births and immigration (12, 25). It not only fails to provide a long-term solution but also raises significant ethical concerns regarding animal welfare. In contrast, sterilization offers a more effective approach as it limits population rebound by preventing birth, so contributes to maintaining reduced population sizes for a longer time. Vaccination and community education also present more sustainable and humane alternatives. These interventions ensure a healthy managed dog population and lower disease transmission, which lowers the economic burden associated with human vaccination and treatment. They could also improve societal well-being by reducing the zoonotic risks (47), or fostering safer communities, potentially benefitting sectors like tourism. Some studies included in this review demonstrated how sterilization combined with either vaccination or responsible ownership was a cost-effective long-term strategy (25, 37, 39, 46), while culling was identified as a costly measure (39). Additionally, free DPM services, such as sterilization, may create dependency within communities and undermine investment in responsible dog ownership practices. Implementing cost-recovery approaches for sterilization, vaccination, mandatory identification and registration, or sheltering could provide a source of income ensuring the sustainability of DPM (15, 44).

The review highlights a range of intervention, impact, and monetary parameters (the costs and benefits) to evaluate the economics of DPM systems, demonstrating the complexity and varied scope of the DPM systems and services. This emphasizes an opportunity to standardize approaches to allow for comparison among studies. Despite this wide range of parameters, we could see similarities in the methods and metrics in the study. The impacts and benefits varied accordingly, with some studies focusing on the societal economic impact (37, 42, 43) while others on the direct economic revenue from the implementation of DPM systems with sheltering dogs (13). However, we observed a lack of focus on the social and non-monetary values associated with dog’s health. In our study, we applied a broad search strategy to avoid missing relevant publications, without restricting to the term “socio-economic,” as its inclusion significantly reduced the initial search results. Despite this, we found only one study (39) that mentioned the loss of a dog’s value due to culling, four studies addressed improved dog welfare (25, 36, 40, 42), and three addressed increased community acceptance of dog populations (40, 42, 47). A study from Noguera et al. (51), discusses this gap in recognizing non-monetary values assigned to animals, particularly in relation to companion animals like dogs (51). There is a need for greater clarity on how we value dogs and their health as well as their broad connections to humans and the environment. Social impacts include the broader range of impacts such as public health, policy and regulations, and overall community well-being reflected by behavioral changes, safety and community perceptions of dogs, and an increase in dog welfare (12, 52). Integrating social aspects in the economics of DPM systems and linking the parameters to human factors might deliver a better understanding of the dog’s value and the overall effectiveness of the DPM systems. Though it might be challenging to quantify these impacts in monetary terms, the overall socio-economic framework will provide a comprehensive evaluation of the DPM system. In a long-term system, the costs and outcomes of the service may occur at different times and build up over the years (53).

Discounting accounts for the changing value of monetary costs and benefits over time, making it particularly relevant for studies conducted over a long period of time (e.g., over multiple years). Although almost all papers consider multiple years in the review, only five accounted for discounted rates, which is a notable gap. The current recommendation is to apply a range of discount rates, including scenarios without discounting, to assess whether the results are influenced by the chosen discounting rate (54, 55). There is also the consideration of how non-monetary health measures such as DALYs should be discounted (56). Heterogeneity in DALY and the burden of disease methods have also hampered the utility and ability of estimates (57). We encourage analysts to consider the recently published reporting guidelines on DALYs to ensure transparency and future utility (58). This approach enhances a more accurate economic evaluation of the DPM services and ensures consistency in decision-making, regardless of the methodological assumptions made about the value of future benefits and costs. Additionally, in the review, economic analysis is often combined with sensitivity analysis to account for uncertainties in the methodologies applied, ensuring that economic outcomes such as CER remain valid under different scenarios or input fluctuations. This strengthens the reliability of economic analysis, allowing for informed decision-making regarding resource allocation in DPM systems.

In this study, the gaps identified were mostly related to data availability and accessibility. In regards to this, data on dog population size or density is crucial in supporting DPM interventions, such as determining the number of sterilizations, vaccinations or adoptions that need to be achieved in a particular region or area (44). For example, without taking in account the baseline dog population size or density in an area, merely the number of sterilizations or vaccinations cannot be used to determine the coverage or success of a DPM service (20). The reliability and credibility of the projected outcomes may be undermined without the integration of objective data (40). This data limitation further poses challenges to estimate the resources required for DPM systems and quantify the impact of DPM services and their relative cost-effectiveness (25). Additionally, the non-monetary benefits and long-term costs and benefits associated with DPM systems were difficult to quantify, making it challenging to address future uncertainties (13). High costs for DPM services, including sterilization and vaccination campaigns, pose difficulties (41, 45, 46), and these challenges also pertain to the sustainability of long-term DPM interventions (38, 39, 44).

During the review process, we came across papers which, despite the presence of quantitative parameters of the DPM systems, lacked data on economic aspects. This makes it challenging to assess the long-term sustainability and financial viability of DPM services. An example is from a study in Greater Bangkok, Thailand to assess the impact of free-roaming dog population (2). This study assessed the intervention parameters such as dogs sterilized, dog density and dog ownership along with the impact on reduction in rabies transmission, increased care and changed perception toward dogs. Similarly, a study for evaluating DPM interventions and their impact in Australia provides information on dog sterilization, microchipping, education on the benefits of DPM and measures impact with vaccination coverage, increase of microchipping in the community, decreased euthanasia, decreased dog attacks, improved community attitudes toward dogs among others (1). Inclusion of additional economic data would have provided a more comprehensive evaluation in these cases. Generally, without economic data, the development of evidence-based policies for the most cost-effective approaches to DPM systems could be hindered, making it challenging to make informed decisions for its implementation. Thus, further research needs prioritizing data gathering and accessibility by organizations or authorities involved in DPM and attributing costs and benefits of the impacts. Developing tools to assist organizations in conducting economic analysis and standardized reporting would benefit DPM evaluation. The tools would ensure consistent methodologies for data collection and economic assessment across different programs. This ensures efficiency, sustainability, and proper resource allocation for implementing DPM services. This effort will ultimately benefit policymakers by facilitating informed decision-making.

Our scoping review has certain limitations that need to be acknowledged. Although we conducted a broad literature with the help of a professional librarian, we may have missed some studies which used different DPM terminologies. Additionally, while we did not apply language restrictions and screened abstracts in other languages (e.g., French and Spanish), it is crucial to note that relevant papers may have been missed due to the search strategy being in English. There was heterogeneity in terminology and the level of context descriptions, we simplified the contexts and terminologies to allow us to group and summarize studies but could have misinterpreted the authors’ meanings.

## 5 Conclusion

Our scoping review identified 14 studies out of more than 7,200 papers that provide insights into the socio-economic assessment of DPM systems, highlighting the costs and benefits of interventions. DPM services may vary with the scope of the system, and this will subsequently affect the intervention, impact and economic parameters, and the methodologies utilized to analyze these parameters. While the economic methods for DPM assessment are generally accessible and straightforward, making them accessible for practical implementation, their application is frequently constrained by the lack of standardized, reliable, and comprehensive data. These limitations arise from insufficient data collection efforts, a lack of standardized methodologies across regions and contexts, and a lack of awareness about the potential value of analyzing economic data. Prioritizing data collection of DPM costs and benefits can lead to future robust economic analysis of the DPM systems. Though challenging to quantify in monetary terms, integrating social aspects in the economics of DPM systems and linking parameters to human factors could enhance the understanding of dogs’ value and overall effectiveness. To achieve this, a standardized framework for data collection and socio-economic assessment would be instrumental in measuring the impact of these DPM systems. This will support effective resource allocation and evidence-based policy making for implementing the most cost-effective and sustainable DPM systems.

## Data Availability

The original contributions presented in the study are included in the article/Supplementary material, further inquiries can be directed to the corresponding author.
