# Supplementary material for: Socio-economic assessment of dog population management systems: a scoping review
Source: Front Vet Sci. 2025 Jan 20;12:1519913. doi: 10.3389/fvets.2025.1519913 (PMC11789200; doi:10.3389/fvets.2025.1519913)
Supplement: Supplementary file 1 [file Supplementary_file_1.docx]

**Annex 1**

The search terms which were used to retrieve the data in PubMed are as follows:

(Population Control[mh] OR Population Dynamics[mh] OR Population Density[mh] OR "population management"[tiab:~7] OR "population control"[tiab:~7] OR "population size"[tiab:~7] OR shelter*[tiab] OR "birth control"[tiab] OR cull*[tiab] OR "fertility control"[tiab] OR "dog population management"[tiab] OR "control measure*"[tiab] OR "control program*"[tiab] OR (program*[tiab] AND "dog control"[tiab]) OR "management program*") AND (Dogs[mh] OR Dogs[tiab] OR Dog[tiab] OR "Canis familiaris"[tiab] OR "canis lupus familiaris*"[tiab] OR "c familiaris"[tiab] OR "c lupus familiaris"[tiab] OR canine[tiab] OR canines[tiab] OR Dog Diseases[mh] OR "dog disease"[tiab] OR "dog diseases"[tiab] OR (dogs[mh] AND ownership[mh]) OR ((dog[tiab] OR dogs[tiab]) AND (ownership[tiab] OR "free roaming"[tiab] OR stray*[tiab] OR ownerless[tiab] OR abandon*[tiab] OR street[tiab] OR roaming[tiab])))
