# Supplementary material for: Socio-economic assessment of dog population management systems: a scoping review
Source: Front Vet Sci. 2025 Jan 20;12:1519913. doi: 10.3389/fvets.2025.1519913 (PMC11789200; doi:10.3389/fvets.2025.1519913)
Supplement: Supplementary file 2 [file Supplementary_file_2.docx]

**Annex 2**

**R codes for UpSet plot**

**install.packages**("UpSetR")

**library**(UpSetR)

**Define the services list**

interventions <- **list**( 
  Smith2022 = **c**("Sterilization", "Culling", "Sheltering", "Awareness"), 
  Cetkovic2022 = **c**("Sterilization", "Sheltering", "Adoption", "Vaccination"), 
  Garde2022 = **c**("Sterilization", "Identification", "Adoption", "Awareness"), 
  Diamante2021 = **c**("Sterilization", "Dog Impounding", "Awareness", "Vaccination"), 
  Larkins2020 = **c**("Sterilization", "Vaccination"), 
  Wallace2017 = **c**("Sterilization", "Awareness", "Vaccination"),  
  Dias2015 = **c**("Sterilization", "Awareness", "Adoption"),  
  Hasler2014 = **c**("Sterilization", "Culling", "Awareness", "Vaccination"),  
  Abbas2014 = **c**("Sterilization", "Awareness", "Vaccination"), 
  Wera2013 = **c**("Culling", "Awareness", "Vaccination"), 
  Hogasen2013 = **c**("Sterilization", "Identification", "Sheltering" ,"Adoption", "Vaccination"), 
  Tenzin2012 = **c**("Sterilization", "Vaccination"), 
  Poss2006 = **c**("Sterilization", "Awareness"),  
  Hasler2012 = **c**("Culling", "Vaccination") 
)

**Get a unique list of all interventions**

all_interventions <- **unique**(**unlist**(interventions))

**Convert the list to a binary matrix**

interventions_binary <- **data.frame**(**matrix**(0, nrow=**length**(interventions), ncol=**length**(all_interventions))) 
**colnames**(interventions_binary) <- all_interventions 
**rownames**(interventions_binary) <- **names**(interventions) 
 
**for** (name **in** **names**(interventions)) { 
  interventions_binary[name, interventions[[name]]] <- 1 
}

#Create the UpSet plot

**upset**(interventions_binary,  
      sets = **colnames**(interventions_binary),  
      order.by = "freq",  
      main.bar.color = "blue", 
      sets.bar.color = "red", 
      keep.order = TRUE, 
      mainbar.y.label = "Intersection Size", 
      sets.x.label = "Set Size")
