# Supplementary material for: Socio-economic assessment of dog population management systems: a scoping review
Source: Front Vet Sci. 2025 Jan 20;12:1519913. doi: 10.3389/fvets.2025.1519913 (PMC11789200; doi:10.3389/fvets.2025.1519913)
Supplement: Supplementary file 3 [file Table_1.docx]

*Supplementary Table 1: Parameters and methods for economic analysis of DPM systems*

| **Study** | **Research approach** | **Observations/Projections timeframe** | **Intervention parameters** | **Impact parameters** | **Monetary costs** | **Societal economic Benefits** | **Discount rates** | **Economic method** | **Economic metrics/outcomes** |
| --- | --- | --- | --- | --- | --- | --- | --- | --- | --- |
| Smith et al, 2022 [18] | Systemic Dynamic Modelling | Projection: 70-year and five-year duration | - Neutering rate - Adoption rate - Sheltering rate - Abandonment rate - Culling rate | - Population reduction of free roaming dogs - Welfare scores for each intervention | - Manpower/Staff costs (Shelter staff to dog ratio: average annual cost): for all six interventions and different coverage rates |  | --- | CEA | Cost per each and combined intervention in terms of staff expenses |
| Ćetković et al., 2022 [9] | Modelling/ Future projections | Projection: 2024 to 2033 (10-year period) | - Number of Adoption - Number of dogs euthanized | - Population reduction of stray dogs - Reduction in dog bites - Reduction in traffic accidents - Reduction in diseases (Echinococcosis, Leishmaniasis) | - Total investment - Salary costs for operating - Long term costs (yearly projections) - Investment costs (land, construction, vehicle procurement, shelter equipment purchase, veterinary equipment, planning and project documentation, and supervision) | - Savings from decrease of dog bites, reduction of traffic road accidents and reduced diseases caused by stray dogs - Revenue generation from shelter (housing owned dogs) ^§^ | 4-5% | CBA  CEA | FBCR, EBCR, FNPV, FIRR, ENPV, EIRR, ENPV  CER: Vet clinic costs per dog (vaccination, sterilization, parasite treatment, other surgical and treatment costs) |
| Garde et al., 2022 [40] | Descriptive analysis of retrospective observational data including legal and quantitative aspects | 2017-2021 (Five years) | - Number of sterilization campaigns - Number of animals sterilized - Dogs microchipped - Number of educational projects implemented |  | - Total budget allocated - Costs of sterilization (including cost of staff) - Costs for community awareness and education |  | --- | CEA | CER (cost per dog sterilized) |
| Diamante et al., 2021 [37] | Retrospective observational data with linear programming modelling | 2011-2018 (9 years) | - Sterilization rate (targeted) - Vaccination coverage - Number of impounded dogs - Number of individuals to attend IEC sessions |  | - Total budget for interventions - Cost of sterilization - Cost of vaccination - Cost of dog impounding - Cost for information and education campaigns |  | --- | CEA | CER (Costs per dog sterilized, vaccinated, costs per dog impounding, costs per IEC session) |
| Larkins et al., 2020 [33] | Retrospective observational data with counterfactual scenario and modelling | 1994/5 – 2016/17 (23-year period) | - Sterilization rate (number sterilized) - Vaccination coverage (Number of dogs vaccinated | - Reduction in roaming dogs’ population - Reduction of dog bites - Decrease in rabies deaths - DALYs averted | - Total costs - Staff costs - Human rabies costs | Savings from reduced dog bites and PEP | 0 and 3% | CBA  CEA | Monetary BCR, Societal BCR, NPV  CER (cost per DALY averted, cost per dog sterilized and vaccinated, cost per dog vaccinated, cost per bite) |
| Wallace et al., 2017 [43] | Predictive with modelling from previous/existing vaccination data, literature review and expert opinions | Baseline: 2015  Projection: 2017 to 2030 (14 years) | - Sterilization rates - Vaccination coverage | - Population reduction of dogs - Improved community health and safety perceptions | - Total dog rabies elimination costs - Sterilization costs - Vaccination costs - Personnel, equipment (vaccine, syringes) and overhead costs for vaccination |  | 3% | CEA | CER (cost per female dog sterilized, cost per dog vaccinated) |
| Dias et al., 2015 [41] | Deterministic mathematical modelling and observational data (census) | Observation from census: (Feb 2012 and June 2013), Projection: 20 years period | - Simulated sterilization rates (number of animals sterilized per gender) /per year - Adoption rate |  | - Total costs for 20 years calculated - Costs for manpower (with campaign costs) |  | --- | CEA | CER (cost per surgical intervention) |
| Häsler et al., 2014 [38] | Mixed-method approach (economic, ethical, epidemiological, and social assessments) and observational data | Baseline: (2002- 2006) Intervention: June 2007 to June 2011 (4 years) | - Number of dogs sterilized - Number of dog bites - Number of dogs vaccinated - Baseline: Number of dogs culled | - Dog population reduction - Reduced rabies deaths in animals and humans - DALYs averted (rabies) - Increase acceptance of dog population - Increased dog welfare | - Total costs of intervention - Costs for sterilization and vaccination - Staff costs - Education costs - Costs for human health - Other administrative and operational costs | From DALYs averted | --- | CEA | CER (Human health cost per bite, DALYs averted per cost unit) |
| Abbas et al., 2014 [34] | Retrospective data and modelling, sensitivity analysis | Retrospective data: 2012, Projection (2012 to 2032) – 20 years period | - Sterilization rates - Vaccination coverage - Number of dog bites and rabies cases |  | - Cost for injectable and oral vaccinations for dogs - Costs for surgical sterilization and injectable sterilization - Costs for manpower included in intervention - Human rabies costs - Costs for community awareness (IEC) |  | --- | CEA | CER (cost per sterilization and vaccination, cost per vaccination, cost per human intervention or dog bite) |
| Wera et al., 2013 [35] | Modelling using data from literature, records and experts | 2000 to 2011 (11 years) | - Number of dogs vaccinated - Number of dogs culled | Loss of dog’s value due to culling | - Total costs of intervention - Vaccination cost - Cost for culling - Human rabies costs - Costs for dog owners |  | 6% | Activity based costing  CEA | CER (Cost per dog vaccinated, cost per dog culled) |
| Høgåsen et al., 2013 [32] | Deterministic Modelling using retrospective data, literature and from local veterinarians | Retrospective data: 2000 to 2006 (11 years)  Projection: 2013 to 2022 (10 years) | - Number of free roaming, kennel, block and stray dogs - Number of adoptions - Number of captures | - Reduction of stray dogs through conversion to Kennel dogs (KD) or Block dogs (BD) - Nuisance index | - Annual direct costs of the program - Costs for dog kennelling (converting SD to KD or BD) | Savings predicted with increased adoption | --- | CBA  CEA | CER (cost for per dog catching, sterilisation, microchipping, vaccination, medication, Leishmania testing, euthanasia)  Cost to convert and maintain each SD to KD or BD |
| Tenzin et al., 2012 [39] | Retrospective data from various governmental and health records, also used for modelling | Retrospective data: 2001-2008 (7 years), Projection: 2012 to 2022 (10 years) | - Dogs sterilized - Dogs vaccinated |  | - Mass dog vaccination costs - Dog sterilization costs - Human rabies costs |  | --- | CEA  CBA | CER (Cost per dog sterilized, cost per dog vaccinated)  Income loss per person per dog vaccinated, societal costs per dog vaccination (direct loss plus income loss) |
| Häsler et al., 2012 [36] | Observational data and modelling | Baseline: October 2010 to March 2011 Projection: 2010 – 2021 (11 years) | - Number of dogs culled - Number of dogs vaccinated and predicted vaccination coverage - Number of dog bites | - Reduction in rabies deaths - DALYs averted - Social acceptability - Animal Welfare | - Total costs of vaccination (including vaccine, operational and staff costs) - Culling costs for baseline - Cost of dog bite burden (human disease costs) - Loss due to reduced tourists | - Benefit from vaccination compared to culling - Savings from PEP and bite treatment | 3.5% | CBA  CEA | NPV  CER (Cost per dog vaccinated; treatment cost per dog bite). ICER |
| Poss and Everett, 2006 [42] | Observational data | 2004 (5 months) | Dogs sterilized |  | - Total cost per session - Staff costs |  | Not Applicable | CEA | CER (Cost per dog sterilized (for voluntary and paid staffs separately) |

*§ This study provides direct revenue from DPM systems*

*CBA: Cost-Benefit Analysis, CEA: Cost-effectiveness Analysis, IEC: Information and Education campaigns, BCR: Benefit-cost Ratio, CER; Cost-effectiveness ratio, ICER: incremental cost-effectiveness ratio, FBCR: Financial-Benefit Cost Ratio, EBCR: Economic Benefit Cost Ratio, NPV: Net Present value, FNPV: Financial Net present value, FIRR: Financial Internal Rate of Return, ENPV: Economic Net Present Value, EIRR: Economic Internal Rate of Return*

*PTRAC: National Program for Responsible Ownership of Companion Animals (PTRAC in Spanish: Programa Tenencia Responsable para Animales de Compañía)*
